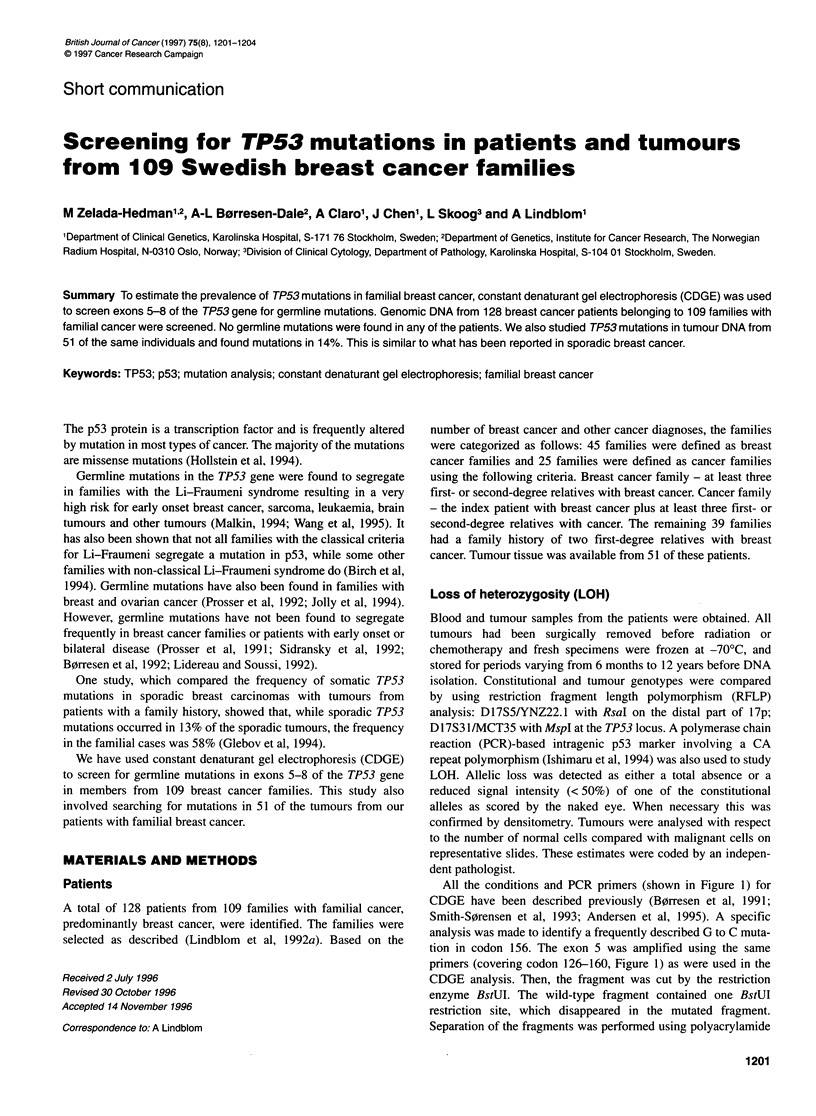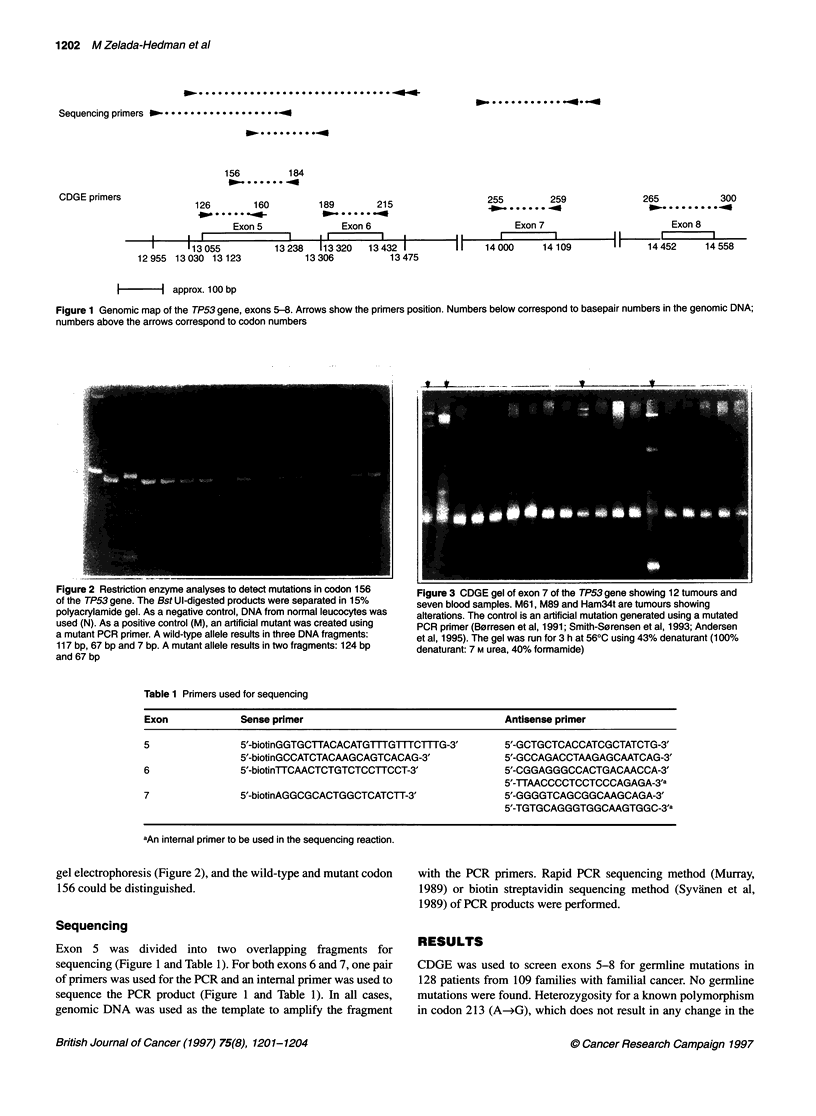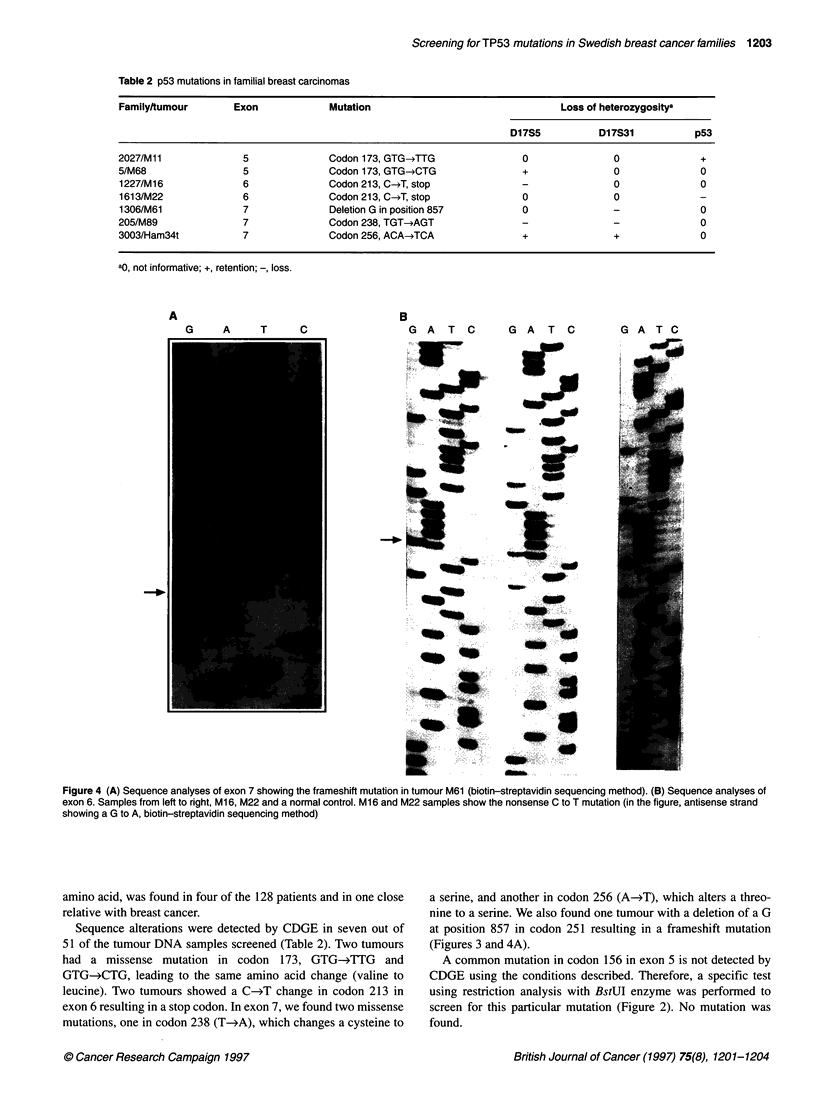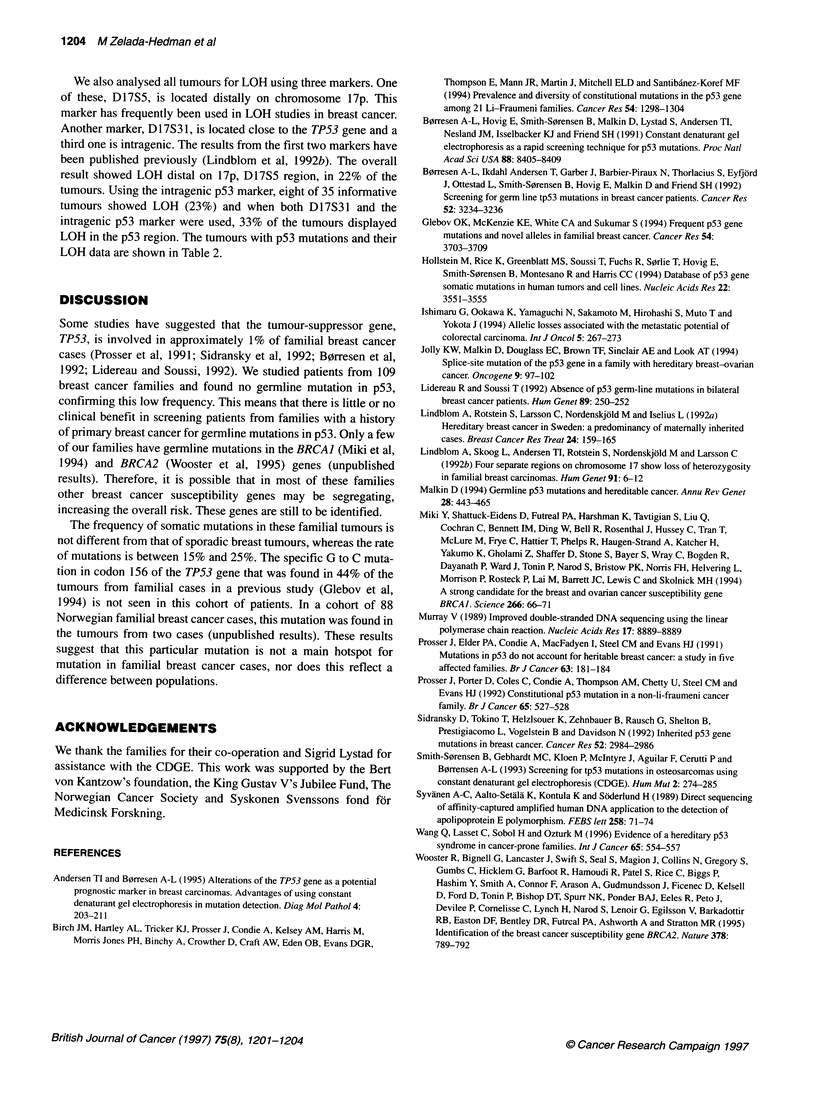# Screening for TP53 mutations in patients and tumours from 109 Swedish breast cancer families.

**DOI:** 10.1038/bjc.1997.205

**Published:** 1997

**Authors:** M. Zelada-Hedman, A. L. BÃ¸rresen-Dale, A. Claro, J. Chen, L. Skoog, A. Lindblom

**Affiliations:** Department of Clinical Genetics, Karolinska Hospital, Stockholm, Sweden.

## Abstract

**Images:**


British Journal of Cancer (1997) 75(8), 1201–1204
© 1997 Cancer Research Campaign

Short communication

### Screening for *TP53* mutations in patients and tumours from 109 Swedish breast cancer families

**M Zelada-Hedman[1,2], A-L Børresen-Dale[2], A Claro[1], J Chen[1], L Skoog[3] and A Lindblom[1]**

[1]Department of Clinical Genetics, Karolinska Hospital, S-171 76 Stockholm, Sweden; [2]Department of Genetics, Institute for Cancer Research, The Norwegian Radium Hospital, N-0310 Oslo, Norway; [3]Division of Clinical Cytology, Department of Pathology, Karolinska Hospital, S-104 01 Stockholm, Sweden.

**Summary** To estimate the prevalence of *TP53* mutations in familial breast cancer, constant denaturant gel electrophoresis (CDGE) was used to screen exons 5–8 of the *TP53* gene for germline mutations. Genomic DNA from 128 breast cancer patients belonging to 109 families with familial cancer were screened. No germline mutations were found in any of the patients. We also studied *TP53* mutations in tumour DNA from 51 of the same individuals and found mutations in 14%. This is similar to what has been reported in sporadic breast cancer.

**Keywords:** TP53; p53; mutation analysis; constant denaturant gel electrophoresis; familial breast cancer

The p53 protein is a transcription factor and is frequently altered by mutation in most types of cancer. The majority of the mutations are missense mutations (Hollstein et al, 1994).

Germline mutations in the *TP53* gene were found to segregate in families with the Li–Fraumeni syndrome resulting in a very high risk for early onset breast cancer, sarcoma, leukaemia, brain tumours and other tumours (Malkin, 1994; Wang et al, 1995). It has also been shown that not all families with the classical criteria for Li–Fraumeni segregate a mutation in p53, while some other families with non-classical Li–Fraumeni syndrome do (Birch et al, 1994). Germline mutations have also been found in families with breast and ovarian cancer (Prosser et al, 1992; Jolly et al, 1994). However, germline mutations have not been found to segregate frequently in breast cancer families or patients with early onset or bilateral disease (Prosser et al, 1991; Sidransky et al, 1992; Børresen et al, 1992; Lidereau and Soussi, 1992).

One study, which compared the frequency of somatic *TP53* mutations in sporadic breast carcinomas with tumours from patients with a family history, showed that, while sporadic *TP53* mutations occurred in 13% of the sporadic tumours, the frequency in the familial cases was 58% (Glebov et al, 1994).

We have used constant denaturant gel electrophoresis (CDGE) to screen for germline mutations in exons 5–8 of the *TP53* gene in members from 109 breast cancer families. This study also involved searching for mutations in 51 of the tumours from our patients with familial breast cancer.

### MATERIALS AND METHODS

### Patients

A total of 128 patients from 109 families with familial cancer, predominantly breast cancer, were identified. The families were selected as described (Lindblom et al, 1992*a*). Based on the number of breast cancer and other cancer diagnoses, the families were categorized as follows: 45 families were defined as breast cancer families and 25 families were defined as cancer families using the following criteria. Breast cancer family – at least three first- or second-degree relatives with breast cancer. Cancer family – the index patient with breast cancer plus at least three first- or second-degree relatives with cancer. The remaining 39 families had a family history of two first-degree relatives with breast cancer. Tumour tissue was available from 51 of these patients.

### Loss of heterozygosity (LOH)

Blood and tumour samples from the patients were obtained. All tumours had been surgically removed before radiation or chemotherapy and fresh specimens were frozen at –70°C, and stored for periods varying from 6 months to 12 years before DNA isolation. Constitutional and tumour genotypes were compared by using restriction fragment length polymorphism (RFLP) analysis: D17S5/YNZ22.1 with *Rsa*I on the distal part of 17p; D17S31/MCT35 with *Msp*I at the *TP53* locus. A polymerase chain reaction (PCR)-based intragenic p53 marker involving a CA repeat polymorphism (Ishimaru et al, 1994) was also used to study LOH. Allelic loss was detected as either a total absence or a reduced signal intensity (< 50%) of one of the constitutional alleles as scored by the naked eye. When necessary this was confirmed by densitometry. Tumours were analysed with respect to the number of normal cells compared with malignant cells on representative slides. These estimates were coded by an independent pathologist.

All the conditions and PCR primers (shown in Figure 1) for CDGE have been described previously (Børresen et al, 1991; Smith-Sørensen et al, 1993; Andersen et al, 1995). A specific analysis was made to identify a frequently described G to C mutation in codon 156. The exon 5 was amplified using the same primers (covering codon 126–160, Figure 1) as were used in the CDGE analysis. Then, the fragment was cut by the restriction enzyme *Bst*UI. The wild-type fragment contained one *Bst*UI restriction site, which disappeared in the mutated fragment. Separation of the fragments was performed using polyacrylamide

*Received 2 July 1996*
*Revised 30 October 1996*
*Accepted 14 November 1996*

*Correspondence to:* A Lindblom

**Figure 1** Genomic map of the *TP53* gene, exons 5–8. Arrows show the primers position. Numbers below correspond to basepair numbers in the genomic DNA; numbers above the arrows correspond to codon numbers

**Figure 2** Restriction enzyme analyses to detect mutations in codon 156 of the *TP53* gene. The *Bst* UI-digested products were separated in 15% polyacrylamide gel. As a negative control, DNA from normal leucocytes was used (N). As a positive control (M), an artificial mutant was created using a mutant PCR primer. A wild-type allele results in three DNA fragments: 117 bp, 67 bp and 7 bp. A mutant allele results in two fragments: 124 bp and 67 bp

**Figure 3** CDGE gel of exon 7 of the *TP53* gene showing 12 tumours and seven blood samples. M61, M89 and Ham34t are tumours showing alterations. The control is an artificial mutation generated using a mutated PCR primer (Børresen et al, 1991; Smith-Sørensen et al, 1993; Andersen et al, 1995). The gel was run for 3 h at 56°C using 43% denaturant (100% denaturant: 7 M urea, 40% formamide)

**Table 1** Primers used for sequencing

| Exon | Sense primer | Antisense primer |
|---|---|---|
| 5 | 5′-biotinGGTGCTTACACATGTTTGTTTCTTTG-3′ | 5′-GCTGCTCACCATCGCTATCTG-3′ |
| | 5′-biotinGCCATCTACAAGCAGTCACAG-3′ | 5′-GCCAGACCTAAGAGCAATCAG-3′ |
| 6 | 5′-biotinTTCAACTCTGTCTCCTTCCT-3′ | 5′-CGGAGGGCCACTGACAACCA-3′ |
| | | 5′-TTAACCCCTCCTCCCAGAGA-3′[a] |
| 7 | 5′-biotinAGGCGCACTGGCTCATCTT-3′ | 5′-GGGGTCAGCGGCAAGCAGA-3′ |
| | | 5′-TGTGCAGGGTGGCAAGTGGC-3′[a] |

[a]An internal primer to be used in the sequencing reaction.

gel electrophoresis (Figure 2), and the wild-type and mutant codon 156 could be distinguished.

### Sequencing

Exon 5 was divided into two overlapping fragments for sequencing (Figure 1 and Table 1). For both exons 6 and 7, one pair of primers was used for the PCR and an internal primer was used to sequence the PCR product (Figure 1 and Table 1). In all cases, genomic DNA was used as the template to amplify the fragment with the PCR primers. Rapid PCR sequencing method (Murray, 1989) or biotin streptavidin sequencing method (Syvänen et al, 1989) of PCR products were performed.

### RESULTS

CDGE was used to screen exons 5–8 for germline mutations in 128 patients from 109 families with familial cancer. No germline mutations were found. Heterozygosity for a known polymorphism in codon 213 (A→G), which does not result in any change in the

© Cancer Research Campaign 1997

**Table 2** p53 mutations in familial breast carcinomas

| Family/tumour | Exon | Mutation | Loss of heterozygosity[a] | | |
|---|---|---|---|---|---|
| | | | D17S5 | D17S31 | p53 |
| 2027/M11 | 5 | Codon 173, GTG→TTG | 0 | 0 | + |
| 5/M68 | 5 | Codon 173, GTG→CTG | + | 0 | 0 |
| 1227/M16 | 6 | Codon 213, C→T, stop | – | 0 | 0 |
| 1613/M22 | 6 | Codon 213, C→T, stop | 0 | 0 | – |
| 1306/M61 | 7 | Deletion G in position 857 | 0 | – | 0 |
| 205/M89 | 7 | Codon 238, TGT→AGT | – | – | 0 |
| 3003/Ham34t | 7 | Codon 256, ACA→TCA | + | + | 0 |

[a]0, not informative; +, retention; –, loss.

**Figure 4** (**A**) Sequence analyses of exon 7 showing the frameshift mutation in tumour M61 (biotin–streptavidin sequencing method). (**B**) Sequence analyses of exon 6. Samples from left to right, M16, M22 and a normal control. M16 and M22 samples show the nonsense C to T mutation (in the figure, antisense strand showing a G to A, biotin–streptavidin sequencing method)

amino acid, was found in four of the 128 patients and in one close relative with breast cancer.

Sequence alterations were detected by CDGE in seven out of 51 of the tumour DNA samples screened (Table 2). Two tumours had a missense mutation in codon 173, GTG→TTG and GTG→CTG, leading to the same amino acid change (valine to leucine). Two tumours showed a C→T change in codon 213 in exon 6 resulting in a stop codon. In exon 7, we found two missense mutations, one in codon 238 (T→A), which changes a cysteine to a serine, and another in codon 256 (A→T), which alters a threonine to a serine. We also found one tumour with a deletion of a G at position 857 in codon 251 resulting in a frameshift mutation (Figures 3 and 4A).

A common mutation in codon 156 in exon 5 is not detected by CDGE using the conditions described. Therefore, a specific test using restriction analysis with *Bst*UI enzyme was performed to screen for this particular mutation (Figure 2). No mutation was found.

*© Cancer Research Campaign 1997*

We also analysed all tumours for LOH using three markers. One of these, D17S5, is located distally on chromosome 17p. This marker has frequently been used in LOH studies in breast cancer. Another marker, D17S31, is located close to the *TP53* gene and a third one is intragenic. The results from the first two markers have been published previously (Lindblom et al, 1992*b*). The overall result showed LOH distal on 17p, D17S5 region, in 22% of the tumours. Using the intragenic p53 marker, eight of 35 informative tumours showed LOH (23%) and when both D17S31 and the intragenic p53 marker were used, 33% of the tumours displayed LOH in the p53 region. The tumours with p53 mutations and their LOH data are shown in Table 2.

### DISCUSSION

Some studies have suggested that the tumour-suppressor gene, *TP53*, is involved in approximately 1% of familial breast cancer cases (Prosser et al, 1991; Sidransky et al, 1992; Børresen et al, 1992; Lidereau and Soussi, 1992). We studied patients from 109 breast cancer families and found no germline mutation in p53, confirming this low frequency. This means that there is little or no clinical benefit in screening patients from families with a history of primary breast cancer for germline mutations in p53. Only a few of our families have germline mutations in the *BRCA1* (Miki et al, 1994) and *BRCA2* (Wooster et al, 1995) genes (unpublished results). Therefore, it is possible that in most of these families other breast cancer susceptibility genes may be segregating, increasing the overall risk. These genes are still to be identified.

The frequency of somatic mutations in these familial tumours is not different from that of sporadic breast tumours, whereas the rate of mutations is between 15% and 25%. The specific G to C mutation in codon 156 of the *TP53* gene that was found in 44% of the tumours from familial cases in a previous study (Glebov et al, 1994) is not seen in this cohort of patients. In a cohort of 88 Norwegian familial breast cancer cases, this mutation was found in the tumours from two cases (unpublished results). These results suggest that this particular mutation is not a main hotspot for mutation in familial breast cancer cases, nor does this reflect a difference between populations.

### ACKNOWLEDGEMENTS

We thank the families for their co-operation and Sigrid Lystad for assistance with the CDGE. This work was supported by the Bert von Kantzow's foundation, the King Gustav V's Jubilee Fund, The Norwegian Cancer Society and Syskonen Svenssons fond för Medicinsk Forskning.

### REFERENCES

Andersen TI and Børresen A-L (1995) Alterations of the *TP53* gene as a potential prognostic marker in breast carcinomas. Advantages of using constant denaturant gel electrophoresis in mutation detection. *Diag Mol Pathol* **4**: 203–211

Birch JM, Hartley AL, Tricker KJ, Prosser J, Condie A, Kelsey AM, Harris M, Morris Jones PH, Binchy A, Crowther D, Craft AW, Eden OB, Evans DGR, Thompson E, Mann JR, Martin J, Mitchell ELD and Santibánez-Koref MF (1994) Prevalence and diversity of constitutional mutations in the p53 gene among 21 Li–Fraumeni families. *Cancer Res* **54**: 1298–1304

Børresen A-L, Hovig E, Smith-Sørensen B, Malkin D, Lystad S, Andersen TI, Nesland JM, Isselbacker KJ and Friend SH (1991) Constant denaturant gel electrophoresis as a rapid screening technique for p53 mutations. *Proc Natl Acad Sci USA* **88**: 8405–8409

Børresen A-L, Ikdahl Andersen T, Garber J, Barbier-Piraux N, Thorlacius S, Eyfjörd J, Ottestad L, Smith-Sørensen B, Hovig E, Malkin D and Friend SH (1992) Screening for germ line tp53 mutations in breast cancer patients. *Cancer Res* **52**: 3234–3236

Glebov OK, McKenzie KE, White CA and Sukumar S (1994) Frequent p53 gene mutations and novel alleles in familial breast cancer. *Cancer Res* **54**: 3703–3709

Hollstein M, Rice K, Greenblatt MS, Soussi T, Fuchs R, Sørlie T, Hovig E, Smith-Sørensen B, Montesano R and Harris CC (1994) Database of p53 gene somatic mutations in human tumors and cell lines. *Nucleic Acids Res* **22**: 3551–3555

Ishimaru G, Ookawa K, Yamaguchi N, Sakamoto M, Hirohashi S, Muto T and Yokota J (1994) Allelic losses associated with the metastatic potential of colorectal carcinoma. *Int J Oncol* **5**: 267–273

Jolly KW, Malkin D, Douglass EC, Brown TF, Sinclair AE and Look AT (1994) Splice-site mutation of the p53 gene in a family with hereditary breast–ovarian cancer. *Oncogene* **9**: 97–102

Lidereau R and Soussi T (1992) Absence of p53 germ-line mutations in bilateral breast cancer patients. *Hum Genet* **89**: 250–252

Lindblom A, Rotstein S, Larsson C, Nordenskjöld M and Iselius L (1992*a*) Hereditary breast cancer in Sweden: a predominancy of maternally inherited cases. *Breast Cancer Res Treat* **24**: 159–165

Lindblom A, Skoog L, Andersen TI, Rotstein S, Nordenskjöld M and Larsson C (1992*b*) Four separate regions on chromosome 17 show loss of heterozygosity in familial breast carcinomas. *Hum Genet* **91**: 6–12

Malkin D (1994) Germline p53 mutations and hereditable cancer. *Annu Rev Genet* **28**: 443–465

Miki Y, Shattuck-Eidens D, Futreal PA, Harshman K, Tavtigian S, Liu Q, Cochran C, Bennett IM, Ding W, Bell R, Rosenthal J, Hussey C, Tran T, McLure M, Frye C, Hattier T, Phelps R, Haugen-Strand A, Katcher H, Yakumo K, Gholami Z, Shaffer D, Stone S, Bayer S, Wray C, Bogden R, Dayanath P, Ward J, Tonin P, Narod S, Bristow PK, Norris FH, Helvering L, Morrison P, Rosteck P, Lai M, Barrett JC, Lewis C and Skolnick MH (1994) A strong candidate for the breast and ovarian cancer susceptibility gene *BRCA1*. *Science* **266**: 66–71

Murray V (1989) Improved double-stranded DNA sequencing using the linear polymerase chain reaction. *Nucleic Acids Res* **17**: 8889–8889

Prosser J, Elder PA, Condie A, MacFadyen I, Steel CM and Evans HJ (1991) Mutations in p53 do not account for heritable breast cancer: a study in five affected families. *Br J Cancer* **63**: 181–184

Prosser J, Porter D, Coles C, Condie A, Thompson AM, Chetty U, Steel CM and Evans HJ (1992) Constitutional p53 mutation in a non-li-fraumeni cancer family. *Br J Cancer* **65**: 527–528

Sidransky D, Tokino T, Helzlsouer K, Zehnbauer B, Rausch G, Shelton B, Prestigiacomo L, Vogelstein B and Davidson N (1992) Inherited p53 gene mutations in breast cancer. *Cancer Res* **52**: 2984–2986

Smith-Sørensen B, Gebhardt MC, Kloen P, McIntyre J, Aguilar F, Cerutti P and Børrensen A-L (1993) Screening for tp53 mutations in osteosarcomas using constant denaturant gel electrophoresis (CDGE). *Hum Mut* **2**: 274–285

Syvänen A-C, Aalto-Setälä K, Kontula K and Söderlund H (1989) Direct sequencing of affinity-captured amplified human DNA application to the detection of apolipoprotein E polymorphism. *FEBS lett* **258**: 71–74

Wang Q, Lasset C, Sobol H and Ozturk M (1996) Evidence of a hereditary p53 syndrome in cancer-prone families. *Int J Cancer* **65**: 554–557

Wooster R, Bignell G, Lancaster J, Swift S, Seal S, Magion J, Collins N, Gregory S, Gumbs C, Hicklem G, Barfoot R, Hamoudi R, Patel S, Rice C, Biggs P, Hashim Y, Smith A, Connor F, Arason A, Gudmundsson J, Ficenec D, Kelsell D, Ford D, Tonin P, Bishop DT, Spurr NK, Ponder BAJ, Eeles R, Peto J, Devilee P, Cornelisse C, Lynch H, Narod S, Lenoir G, Egilsson V, Barkadottir RB, Easton DF, Bentley DR, Futrcal PA, Ashworth A and Stratton MR (1995) Identification of the breast cancer susceptibility gene *BRCA2*. *Nature* **378**: 789–792

© Cancer Research Campaign 1997